# Extrapleural Closure of Patent Ductus Arteriosus: How We Do It

**DOI:** 10.21470/1678-9741-2019-0473

**Published:** 2020

**Authors:** Nicola Pradegan, Ysailis Mariñez Muñoz, Vladimiro L. Vida, Juan R. Leon-Wyss

**Affiliations:** 1 Pediatric Cardiac Surgery Unit, CEDIMAT Cardiovascular Center, Santo Domingo, Dominican Republic.; 2 Department of Cardiac Thoracic, Cardiac Surgery Unit, Vascular Sciences and Public Health, Padua University Hospital, Padova, Italy.

**Keywords:** Infant, Newborn, Ductus Arteriosus, Patent, Thoracic Duct, Recurrent Laryngeal Nerve, Birth Rate, Operative Time, Developing Countries, Ligation, Pleural Cavity

## Abstract

Patent ductus arteriosus (PDA) is a clinical condition mostly found in premature newborns. Among several medical, surgical and interventional treatment options, extrapleural ligation through a left minithoracotomy is recognized as a safe, efficient and less expensive technique. In fact, it requires short surgical times, grants good exposure of the duct and nearby structures (*e.g.*, thoracic duct, left recurrent laryngeal nerve), and avoids pleural space opening and subsequent pulmonary complications in preterm patients. This approach seems ideal due to its lower costs, especially in developing countries with a high birth rate and limited resources.

**Table:** 

Abbreviations, acronyms & symbols	
**PDA**	**= Patent ductus arteriosus**

## INTRODUCTION

Patent ductus arteriosus (PDA), a persistent communication between the descending thoracic aorta and the pulmonary artery resulting from failure of normal physiological closure of the fetal ductus, is a rare condition, typically found in premature infants (50%). Its closure is required when medical therapy fails (*e.g*., nonsteroidal anti-inflammatory drugs) to prevent chronic left ventricular volume overload and pulmonary hypertension that lead to the development of pulmonary vascular obstructive disease. Among different mini-invasive strategies that have emerged in the last decades (percutaneous closure with intravascular coils or plugs, video-assisted thoracoscopic closure)^[1,2]^, extrapleural ligation through a muscle-sparing left minithoracotomy has been recognized as a safe, efficient and less expensive technique^[3-6]^. We aimed to show the technical approach of a mini-invasive extrapleural PDA closure.

### Technique

**Fig. 1:**
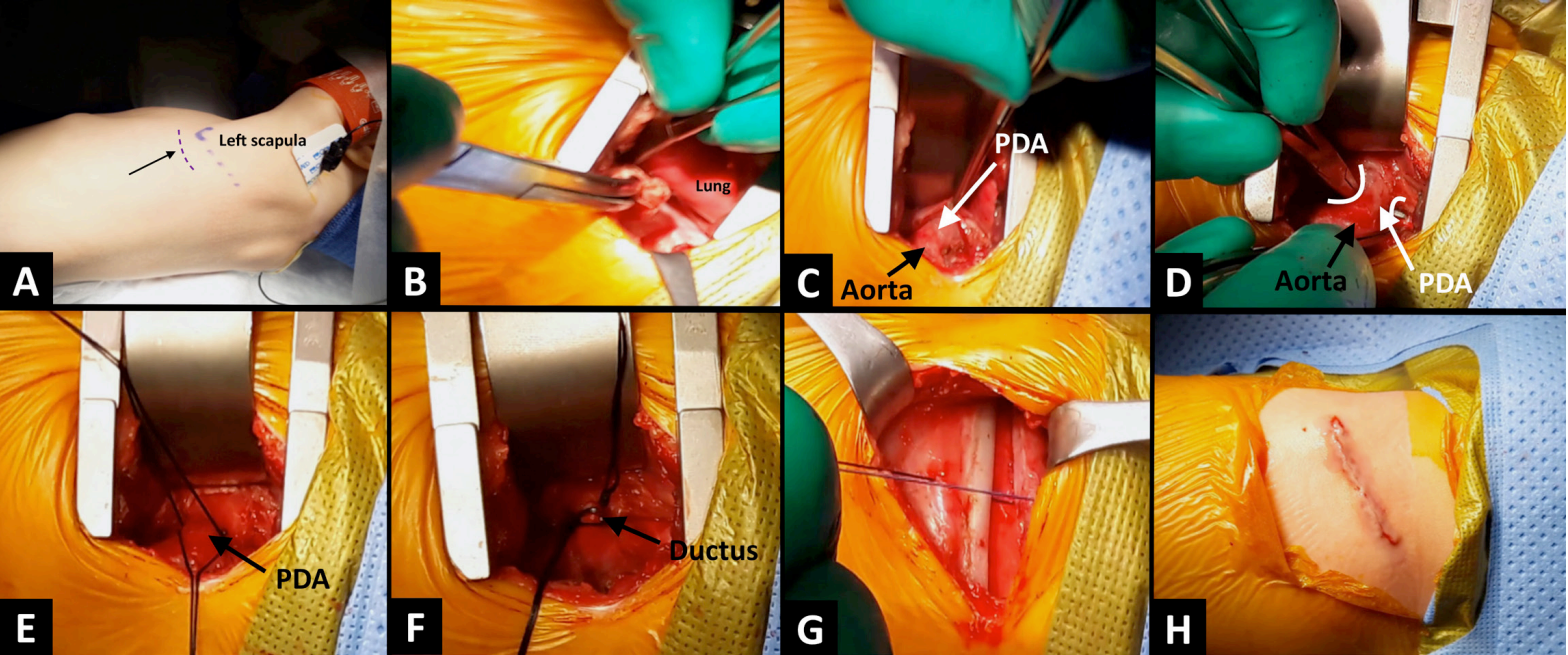
A) left subscapular incision site (black arrow). B) opened IV intercostal space and virtual area between the chest and the parietal pleura, which is gently dissected by means of a small gauze ball. C) descending aorta and patent ductus arteriosus (PDA) carefully dissected. D) Mixter right angle forceps around the duct, after visualizing and avoiding the left recurrent laryngeal nerve. E) the duct is encircled with two silk sutures. F) duct doubly ligated and clipped with a titanium clip. G) intercostal space closure after pulmonary re-expansion, without the need for the placement of a temporary chest tube. H) incision closed.

Under general anesthesia and endotracheal intubation, the patient is placed in a right lateral decubitus position. Through a limited subscapular skin- and muscle-sparing incision (Figure 1A), the superficial thoracic fascia muscularis is open. The periosteum of the fifth rib is then incised, and the parietal pleura is detached with careful dissection from the thoracic wall through the IV intercostal space (Figure 1B). The left lung is partially retracted, and the aorta is exposed. The PDA is dissected from the surrounding tissues with the help of a Mixter right angle forceps (Figures 1C and 1D). 

After identifying the thoracic duct and the left recurrent laryngeal nerve, the duct is encircled by two silk sutures (Figure 1E) and then doubly ligated, leaving sufficient space between the two ligatures to allow the placement of a 10-mm titanium clip to ensure triple duct occlusion (Figure 1F). After adequate hemostasis and excluding an accidental tear of the visceral pleura, the lung is expanded to prevent air retention within the extrapleural space. The chest is then closed without the use of drainage (Figures 1G and 1H) (Video).

## DISCUSSION

PDA closure can be achieved with different approaches^[1-6]^. Nevertheless, in the past 10 years, there has been a growing interest in percutaneous treatment (occluding devices), which seem to show lower rates of complications^[7]^. However, they have been used mainly in patients >2 kg. Consequently, according to our large experience^[3]^, in the era of emerging pediatric minimally invasive surgery^[8]^, extrapleural PDA repair has been recognized for its outstanding results with a low rate of complications in selected patients. Technically, this strategy requires short surgical times (<60 minutes), guarantees good surgical exposure of the PDA (also in patients <1.5 kg who are not routinely managed through catheterization), offering direct visualization of the thoracic duct and of the left recurrent laryngeal nerve, thus preventing complications secondary to its damage. In addition, an intact pleural space reduces the risk of pulmonary complications (*e.g*., surgical injury, pleural effusion, pneumothorax), a major concern especially in premature patients who usually suffer from pulmonary dysplasia. Even though a recent study has stated that a sternotomy approach would be safer for lung protection in preterm patients^[9]^, we still think that reaching the PDA while preserving the parietal pleura has additional advantages. This technique also provides excellent aesthetical results. Although percutaneous strategies are growing in the field of PDA repair, there are still published concerns about the higher risk of residual shunt and the greater possibility of device migration or embolization, especially in low-weight preterm patients^[10]^. This extrapleural surgical approach is significantly cost-saving in comparison to other strategies, thus being a suitable approach especially in underdeveloped countries^[3]^. Even if technically achievable, we do not recommend it in patients >20 kg; in fact, above this weight the parietal pleura is more attached, possibly leading to a higher risk of residual extrapleural hematoma.

## CONCLUSION

The extrapleural surgical repair of PDA is technically effective, safe, with excellent cosmetic results and low-cost procedure, remaining as an important and sometimes preferred approach in the era of 'percutaneous' therapy.

**Table:** 

Authors' roles & responsibilities	
NP YMM VLV JRLW	Substantial contributions to the conception or design of the work; drafting the work or revising it critically for important intellectual content; final approval of the version to be published Acquisition, analysis, or interpretation of data for the work; final approval of the version to be published Drafting the work or revising it critically for important intellectual content; final approval of the version to be published Substantial contributions to the conception or design of the work; drafting the work or revising it critically for important intellectual content; final approval of the version to be published
